# Reproductive technologies, female infertility, and the risk of imprinting-related disorders

**DOI:** 10.1186/s13148-020-00986-3

**Published:** 2020-12-11

**Authors:** Patricia Fauque, Jacques De Mouzon, Aviva Devaux, Sylvie Epelboin, Marie-José Gervoise-Boyer, Rachel Levy, Morgane Valentin, Géraldine Viot, Arianne Bergère, Claire De Vienne, Philippe Jonveaux, Fabienne Pessione

**Affiliations:** 1 CHU Dijon Bourgogne, Laboratoire de Biologie de la Reproduction - CECOS - Université Bourgogne Franche-Comté - INSERM UMR1231, Dijon, France; 2 Unilabs, direction médicale, Clichy-La-Garenne, France; 3 Centre d’assistance medicale à la procreation, biologie de la reproduction, CHU Amiens, Amiens, France; 4 Centre d’assistance medicale à la procreation, gynécologie obstétrique, médecine de la reproduction, Université Paris 7 Diderot, groupe hospitalier Bichat Claude-Bernard, AP–HP, Paris, France; 5 Service de medecine et biologie de la reproduction, Hôpital Saint-Joseph, Marseille 8, France; 6 Sorbonne Université, Saint Antoine Research Center, INSERM équipe Lipodystrophies genetiques et acquises, Service de biologie de la reproduction-CECOS, Hôpital Tenon, AP-HP, 75012 Paris, France; 7 Diagnostic antenatal, gynécologie obstétrique, Université Paris 7 Diderot, groupe hospitalier Bichat Claude-Bernard, AP–HP, Paris, France; 8 Unité de Génétique Clinique de La Muette, 50 rue Nicolo, 75116 Paris, France; 9 Agence de la Biomédecine, 1 avenue du stade de France, 93212 La Plaine Saint Denis, France

**Keywords:** Epigenetic diseases, Female infertility, Assisted reproductive technologies, Children, Singletons

## Abstract

**Background:**

Epidemiological studies suggest that singletons born from assisted reproductive technologies (ART) have a high risk of adverse perinatal outcomes, specifically for imprinting disorders. Because ART processes take place at times when epigenetic reprogramming/imprinting are occurring, there is concern that ART can affect genomic imprints. However, little is currently known about the risk of imprinting defects according to the type of ART or the type of underlying female infertility.

From the French national health database, a cohort of 3,501,495 singletons born over a 5-year period (2013–2017) following fresh embryo or frozen embryo transfers (fresh-ET or FET from in vitro fertilization), intrauterine insemination, or natural conception was followed up to early childhood. Based on clinical features, several syndromes/diseases involving imprinted genes were monitored. The effects of ART conception and the underlying cause of female infertility were assessed.

**Results:**

Compared with infants conceived naturally, children born after fresh-ET had a higher prevalence of imprinting-related diseases, with an aOR of 1.43 [95% CI 1.13–1.81, *p* = 0.003]. Namely, we observed an increased risk of neonatal diabetes mellitus (1.96 aOR [95% CI 1.43–2.70], *p* < 0.001). There was an overall independent increase in risk of imprinting diseases for children with mothers diagnosed with endometriosis (1.38 aOR [95% CI 1.06–1.80], *p* = 0.02). Young and advanced maternal age, primiparity, obesity, smoking, and history of high blood pressure or diabetes were also associated with high global risk.

**Conclusions:**

This prospective epidemiological study showed that the risk of clinically diagnosed imprinting-related diseases is increased in children conceived after fresh embryo transfers or from mothers with endometriosis. The increased perturbations in genomic imprinting could be caused by controlled ovarian hyperstimulation and potentially endometriosis through the impairment of endometrial receptivity and placentation, leading to epigenetic feto-placental changes. Further studies are now needed to improve understanding of the underlying molecular mechanisms (i.e. genetic or epigenetic causes).

## Background

Assisted reproductive technologies (ART), mostly intrauterine insemination (IUI) and in vitro fertilization (IVF), have helped many couples to overcome infertility. Worldwide, millions of children have been born via ART, and they now account for > 4% of births in some European countries [1].

Even though most of these children are considered healthy, there is increasing awareness about the potential consequences of ART on a number of complications potentially linked to epigenetic deregulation [2].

Epidemiological studies suggest that singletons born following the use of ART have an increased risk of adverse perinatal outcomes (e.g. low birth weight after fresh embryo transfers and abnormal placentation) [3, 4]. Furthermore, ART-conceived offspring also have an increased risk of rare imprinting disorders, such as Beckwith–Wiedemann (BWS), Russell–Silver (SRS), Angelman (AS) and Prader–Willi (PWS) syndromes [5–10]. However, there are considerable differences in the reported relative risks (Additional file 1: Table S1), which can mostly be explained by the substantial methodological heterogeneity (findings mostly obtained from voluntary registries or relatively small populations). The systematic reviews on this field have nevertheless made it possible to assess a substantial number of children. In a review, Vermeiden and Bernardus estimated that the birth of a child with BWS is significantly associated with ART, with a pooled relative risk of 5.2 [95% CI 1.6–7.4] [11]. Similarly, a systematic review demonstrated that the combined odds ratio of any imprinting syndromes in children conceived by ART is 3.67 [95% CI 1.39–9.74] when compared with naturally conceived children [12]. In a recent meta-analysis, a positive association was still found between conception after ART and four imprinting conditions (BWS, SRS, AS, PWS), among which BWS had a summary odds ratio of 5.8 [95% CI 3.1–11.1] [13]. It is difficult to draw any conclusions concerning SRS because it is extremely rare [11], but a positive association with ART treatment is likely [13]. No significant associations were found between the incidence of AS or PWS and IVF treatments [11]. Fertility problems could be involved in these two last syndromes in ART children [11], but the results of any meta-analysis must be interpreted in light of the limitations of the contributing studies.

During the periconceptional period, genome-wide epigenetic reprogramming occurs [14, 15]. This reprogramming includes imprinting, which is crucial for the proper development and future health of offspring. The many manipulations and processes of ART (e.g. hormonal stimulation, embryo manipulation, culture and cryopreservation) are concurrent with epigenetic reprogramming and imprinting (i.e. during female gametogenesis and preimplantation embryo development), leading to concerns that the ART themselves could negatively affect epigenetics and the establishment/maintenance of genomic imprints. Importantly, contrary to the molecular aetiologies of BWS children conceived naturally, almost all ART-conceived BWS typically occurred though loss of epigenetic marks in imprinting control regions (close to 95% of children with BWS born after IVF/ICSI vs 50% in the general population) [11]. However, the infertility/subfertility status of the parents may also play a role in the increased incidence of these disorders, as underlined in a Dutch study performed in families with a child with BWS, PWS, or AS [9]. Interestingly, there is increasing evidence that some female infertility syndromes such as polycystic ovary syndrome (PCOS) [16] or endometriosis [17] are associated with epigenetic alterations.

However, so far, the risk of imprinting-related diseases in relation to specific types of ART or underlying causes of female infertility has not been assessed. Therefore, the first aim of this extensive national cohort study was to compare the prevalence of imprinting-related disorders in singletons born after fresh (fresh-ET) or frozen (FET) embryo transfers, intrauterine insemination (IUI), or following natural conception (NC). Our second aim was to study the role of the three major types of female infertility (i.e. endometriosis, PCOS and primary ovarian insufficiency [POI]) on the prevalence of these imprinting-related diseases.

## Results

### Influence of the mode of conception

According to the mode of conception, the prevalence of imprinting-related diseases was 0.10% after NC (*N* = 3478), 0.16% (*N* = 72) after fresh-ET, 0.12% (*N* = 23) after FET, and 0.11% (*N* = 22) after IUI (for a total of 0.14% after ART). Compared with NC infants, children born after fresh-ET had a significantly higher prevalence of imprinting diseases, with an aOR of 1.43 [95% CI 1.13–1.81], *p* = 0.003, whereas no relevant differences were observed for the IUI and the FET groups. After taking into account female infertility status in multivariate analysis, the risk remained significant in the fresh-ET group (aOR of 1.32 [95% CI 1.04–1.69], *p* = 0.02) (Table 1).

**Table 1:** Risk of imprinting-related diseases according to mode of conception and type of female infertility in multivariate analysis

	Total children	Children with at least one imprinting-related disease		Adjusted OR^a^	95% CI		*p*
	*N*	*N*	%				
Mode of conception							
NC	3,417,089	3478	0.10	1			
Fresh-ET	45,303	72	0.16	1.32	1.04	1.69	0.02
FET	18,885	23	0.12	1.04	0.69	1.57	0.86
IUI	20,218	22	0.11	0.97	0.63	1.47	0.87
Female infertility							
Endometriosis	37,398	59	0.16	1.38	1.06	1.80	0.02
PCOS	6977	12	0.17	1.35	0.76	2.39	0.30
POI	1411	5	0.35	2.42	0.99	5.91	0.05

*CI* confidence interval, *Fresh-ET* fresh embryo transfer, *FET* frozen embryo transfer, *IUI* intrauterine insemination, *NC* natural conception, *OR* odds ratio, *PCOS* polycystic ovary syndrome, *POI* primary ovarian insufficiency

^a^Analyses were adjusted for maternal parameters: age, primiparity, smoking, obesity, history of high blood pressure or diabetes, and newborn sex

More specifically, in univariate analysis, we observed a significant increase in the number of cases of neonatal diabetes mellitus (NDM) in the fresh-ET group (*N* = 38, 0.08%) compared with the NC group (*N* = 1438, 0.04%, *p* < 0.001) (Table 2). The increased risk of NDM was confirmed in multivariate analysis (1.96 aOR [95% CI 1.43–2.70], *p* < 0.001), even when the type of female infertility was taken into account (1.58 aOR [95% CI 1.13–2.21], *p* = 0.008) (Table 3). The increased risk of NDM also remained significant after adjustment for gestational diabetes (data not shown). In addition, when analyses were performed on a subset of NDM newborns who needed medication or were hospitalized for diabetes within one year after birth (*n* = 130, corresponding to incidence of 3.7/100,000), the fresh-ET group remained at a higher risk than the NC group (Additional file 1: Table S2).

**Table 2:** Comparison of observed to expected cases for each clinically diagnosed imprinting-related disease according to the type of reproductive technology using univariate analyses (Poisson regression model)

ICD-10 codes	Imprinting-related diseases^a^	Fresh-ET			FET			IUI		
		Observed cases	Expected^b^ cases	*p*	Observed cases	Expected cases	*p*	Observed cases	Expected cases	*p*
Q93.5	AS	8	10	0.62	4	4	0.99	4	4	0.90
Q87.3	BWS	8	5	0.18	0			2	2	0.88
P70.2	NDM	38	19	< 0.001	10	8	0.50	11	9	0.42
C74.9	Neuroblastoma	2	3	0.57	1	1	0.82	0		
E20.1	Pseudohypoparathyroidism	0			0			1	0	0.01
C69.2	Retinoblastoma	3	3	0.86	1	1	0.90	1	1	0.85
Q87.1	SRS/PWS	18	14	0.33	10	6	0.10	4	6	0.35
Q99.8	upd(14)	24	17	0.07	8	7	0.68	10	7	0.34

*AS* Angelman syndrome, *BWS* Beckwith–Wiedemann syndrome, *Fresh-ET* fresh embryo transfer, *FET* frozen embryo transfer, *IUI* intrauterine insemination, *NDM* neonatal diabetes mellitus, *PWS* Prader–Willi syndrome, *SRS* Silver–Russell syndrome, *Upd* uniparental disomy

^a^Diseases based on clinical features and associated to the corresponding ICD-10 code. Some children could have more than one disease diagnosed

^b^Expected cases from NC data

**Table 3:** Risk of NDM after ART and according to the type of female infertility in multivariate analysis

	Total children	Children with NDM		Adjusted OR^a^	95% CI		*p*
	*N*	*N*	%				
Mode of conception							
NC	3,417,089	1438	0.04	1			
Fresh-ET	45,303	38	0.08	1.58	1.13	2.21	0.008
FET	18,885	10	0.05	1.04	0.56	1.96	0.89
IUI	20,218	11	0.05	1.10	0.61	2.00	0.76
Female infertility							
Endometriosis	37,398	28	0.07	1.47	1.00	2.16	0.05
PCOS	6977	6	0.09	1.39	0.62	3.13	0.42
POI	1411	2	0.14	1.93	0.47	7.86	0.36

*CI* confidence interval, *Fresh-ET* fresh embryo transfer, *FET* frozen embryo transfer, *IUI* intrauterine insemination, *NC* natural conception, *OR* odds ratio, *PCOS* polycystic ovary syndrome, *POI* primary ovarian insufficiency

^a^Analyses were adjusted for maternal parameters: age, primiparity, smoking, obesity, history of high blood pressure, or diabetes

### Influence of maternal parameters

According to the type of female infertility, the prevalence of imprinting-related diseases was 0.16% (*N* = 59), 0.17% (*N* = 12), and 0.35% (*N* = 5) for singletons conceived with mothers diagnosed with endometriosis, PCOS and POI, respectively.

**Fig. 1:**
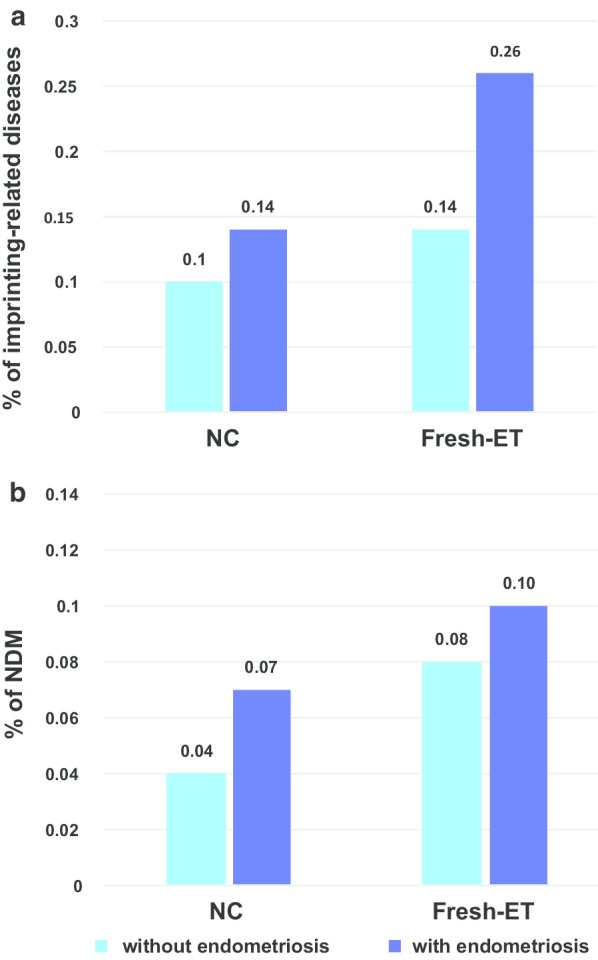
Prevalence of clinically diagnosed imprinting-related diseases (**a**) and NDM (**b**) for children with mothers diagnosed with endometriosis or not according to the mode of conception. *Fresh-ET* fresh embryo transfer, *FET* frozen embryo transfer, *NC* natural conception, *NDM* neonatal diabetes mellitus

There was an overall independent increase in the risk of imprinting-related diseases for the children of mothers with endometriosis (1.38 aOR [95% CI 1.06–1.80], *p* = 0.02) (Table 2). The prevalence of imprinting-related diseases was 0.26% in the fresh-ET group with endometriosis vs 0.14% in the group without endometriosis (Fig. 1). The children of women with POI tended to have higher risk than those from women without POI (2.24 aOR [95% CI 0.99–5.91], *p* = 0.05), but PCOS did not appear to influence the risk of imprinting disorders (Table 1). Specifically, except in the IUI group, the prevalence of NDM was higher in the children whose mothers had endometriosis (Fig. 1). In multivariate analysis, the risk of NDM tended to be higher for children conceived by women with endometriosis (1.47 aOR [95% CI 1.00–2.16], *p* = 0.05) (Table 3). Furthermore, the incidence of children requiring diabetic treatment/hospitalization within one year after birth was higher for women with endometriosis or POI (Additional file 1: Table S2).

Moreover, the other tested maternal factors (i.e. primiparity, obesity, smoking, and history of HBP or diabetes, with significant differences between groups of children, Additional file 1: Table S3) were significantly associated with a higher overall risk of imprinting-related diseases in the multivariate analysis (Table 4). The youngest and advanced-maternal-age women were also at high risk (Table 4).

**Table 4:** Risk of clinically diagnosed imprinting-related diseases for adjustment variables in multivariate analysis

	Adjusted OR^a^	95% CI		*p*
Maternal age (y)				
< 20	1.25	1.03	1.52	0.03
20–29	0.96	0.90	1.03	0.29
30–39	1			
≥ 40	1.58	1.38	1.82	< 0.001
Primiparity	1.28	1.19	1.37	< 0.001
Obesity	1.40	1.23	1.60	< 0.001
Maternal smoking	1.24	1.07	1.43	0.005
History of HBP	4.45	3.69	5.36	< 0.001
History of diabetes	1.68	1.28	2.20	< 0.001
Sex of the newborn				
Female	1			
Male	1.13	1.06	1.20	< 0.001

*95% CI* confidence interval, *HBP* high blood pressure, *OR* odds ratio, *y* years

^a^Analyses were adjusted for infertility status and mode of conception

## Discussion

This unique and large cohort follow-up study made it possible to determine that the risk of imprinting-related disorders was 43% higher in children conceived through IVF or ICSI cycles after fresh embryo transfers than for naturally conceived children. Even though our findings are mostly consistent with the results of a previous review and meta-analysis, we found that the risks are much lower [11, 13]. In addition, we found that female infertility resulting from endometriosis could play a role in the increased risk. Overall, the risks of imprinting-related diseases were mainly observed after treatments involving controlled ovarian hyperstimulation (COH).

Similar to our observations for imprinting diseases, previous studies have reported a reduced risk of several birth defects (e.g. cardiovascular, musculoskeletal, and urogenital abnormalities) following embryo cryopreservation compared with fresh-embryo transfer, either significantly [18] or as a trend [19, 20]. One possible explanation for the reduced risk of imprinting defects following cryopreservation is that the endometrium is not exposed to high levels of gonadotrophins, which may potentially impair trophoblastic invasion, notably through epigenetic changes, and contribute to placental dysfunction and the pathophysiology of disease [3, 21, 22]. Furthermore, no risk of imprinting diseases was associated with intrauterine insemination in our cohort of children. However, by showing that the overall prevalence of congenital malformations was increased with increasing time to naturally-obtained pregnancy, Zhu and colleagues were the first to signal a possible association between infertility and congenital malformations [23]. The notion that infertility itself is a risk factor for increased incidence of birth defects has also been put forward by other researchers [18, 24].

Previous epidemiologic studies have shown that ART-conceived offspring could be at increased risk for rare imprinting disorders, for instance Beckwith–Wiedemann syndrome, with an estimated relative risk of 5.2 [95% CI 1.6–7.4] [11]. However, for most of the population-based studies exploring BWS, the risk was established from voluntary registries [5, 7, 8, 25] and few were based on complete national registries [6, 26, 27] (Additional file 1: Table S1). In addition, among nationwide registries, two were based on imprinting disorders registries (i.e. Gicquel et al. in France [6]; Kallen et al. in Sweden [26]), and one was based on the Danish registries from a relatively small number of ART children (6052 children born after IVF/ICSI) [27].

Therefore, even though our study presents some weaknesses, such as the inability to take into account male infertility, the type of in vitro fertilization method, and the embryo stage at transfer, it also has several strengths. It includes a large cohort of singleton children, it is not restricted to the four imprinting disorders, and it takes into account multiple potential maternal confounding factors. Another strength of the current study is the use of a single national population registry with ascertainment of defects from birth to a child's first birthday or later. However, for some extremely rare syndromes, the number of cases may be too small reliably conclude that there was no difference between groups. Likewise, for childhood cancers involving imprinted genes (i.e. retinoblastoma or neuroblastoma) even though our findings were in line with the literature (Additional file 1: Table S1), longer follow-up is needed to be certain of the absence of risk after ART. Finally, the recorded disorders in our national health databases are based on diagnostic features. The data does not in any way correspond to a molecular diagnosis, which is the only way to identify the molecular mechanism involved in the aetiology of the disorder. However, it is unlikely that our findings are due to biases in reporting or follow-up, which are completely independent from the mode of conception.

In view of the results obtained in this comprehensive cohort follow-up study, we propose that underlying maternal infertility, particularly due to endometriosis, could also contribute to the increased risk of imprinting-related disorders associated with ART. We determined that this risk was 38% higher in children whose mothers were diagnosed with endometriosis than in children whose mothers did not have endometriosis. Further research is needed to evaluate whether the classification of endometriosis or its association with adenomyosis has a specific effect on this risk. Interestingly, some evidence suggests that epigenetics plays a role not only in the development of endometriosis–adenomyosis lesions but also in the heritability of the condition [28].

Epigenetic processes control numerous major cellular functions that occur during development, including changes in gene expression. Most autosomal genes are expressed from both alleles, but imprinted genes are preferentially expressed from alleles inherited from mothers or fathers. The parent-specific expression of imprinted genes is directed by epigenetic marks (mainly DNA methylation), acquired in a sex-specific manner in gametes on regulatory sequences [29]. Then, after fertilization, these parental imprinting marks are protected to avoid global embryo epigenetic reprogramming/demethylation [14]. Imprinted genes, even though they account for small number of human genes, exert crucial roles in embryonic/foetal development including placentation [30], postnatal metabolic pathways, and behaviour associated with the control of resources [31]. Other novel findings reported in the current study are that maternal features (i.e. smoking, obesity, history of HBP or diabetes) were independently associated with an increased risk of imprinting-related diseases. Remarkably, primiparity and young and advanced maternal age also appeared to increase risk. In accordance with our observations, septo-optic dysplasia, which is rarely linked to a genetic cause, has also been associated with primiparity, young maternal age and prenatal exposure to smoking [32, 33]. The underlying aetiology of this condition is likely nutritional or environmental, suggesting the potential role of epigenetic factors [34]. Another hypothesis for this increased risk in the children of young mothers is that young women may be less likely to take vitamins during the periconceptional period, especially folic acid supplementation which known to be fundamental in cellular biosynthesis and DNA methylation pathways [35, 36]. Concerning advanced maternal age, Hara-Isono et al. recently reported that in Japan ART performed on mothers aged ≥ 30 is likely to facilitate epimutation [37]. However, they could not statistically determine whether the effect was caused by ART or the confounding effect of advanced maternal age at conception.

These statements reinforce the concept that the maternal environment can affect foetal development, likely through the effect on the regulation of imprinted genes, primarily in the placenta [2]. We can speculate that the control of these maternal parameters could preserve the appropriate early regulation of imprinting, which could potentially reduce diseases.

Interestingly, NDM was the condition that was most strongly associated with fresh embryo transfer and endometriosis in our results. NDM, which is characterized by hyperglycaemia in the neonatal period, associates growth retardation and hypoglycaemia. It is called transient (TNDM) when it remits during infancy (in about half of children affected by hyperglycaemia), and recovery occurs before 18 months in approximately 50% of these infants. However, infants with TNDM are more prone to developing type 2 diabetes later in life. The dysregulation of two imprinted genes (*PLAGL1* and *HYMAI*) involved in most of the cases is a probable mechanism. These genes are located at the 6q24 region. *PLAGL1* encodes a zinc finger protein which controls cell cycle and apoptosis and enhances insulin secretion, functioning as a regulator of pancreatic β-cells [38, 39]. Furthermore, *PLAGL1* and *HYMAI* are also described as the main regulators of the imprinted network involved in cellular growth and metabolism [40, 41]. TNDM results from a ‘double dose’ of the paternal epigenotype (a twofold overexpression of *PLAGL1/HYMAI*). In the general population, paternal uniparental disomy (patUPD6) is involved in 40% of cases, paternal chromosome duplication in 32% of cases, and hypomethylation at the maternal allele of 6q24 region in 28% [39].

To our knowledge, the underlying molecular mechanism of NDM after ART has not been specifically studied, but, like for BWS, the loss of methylation might be more frequent after ART. Further studies are needed to establish whether the deregulation of imprinted genes that occurs in ART children with NDM is caused by epigenetic or genetic mechanisms. All of the imprinting-related disorders studied here could potentially be linked to genetic abnormalities, but only the results of such further studies could shed light on the underlying molecular mechanisms, making it possible to develop clinical prevention strategies.

Overall, this large population-based study shows that the risk of imprinting diseases, especially neonatal diabetes mellitus, is increased in children conceived after fresh embryo transfers or from mothers with endometriosis. One speculative explanation for this observation is that controlled ovarian hyperstimulation, and thus potentially endometriosis, induces perturbations in genomic imprinting by impairing endometrial receptivity and placentation, leading to epigenetic feto-placental changes. Vast evidence suggests that the gestational environment has an impact on epigenetic mechanisms including imprinting, which can lead to harmful outcomes in the offspring [2].

These novel findings highlight the importance of taking into account the mode/process of conception and the type of infertility.

## Methods

### Data sources

This study was piloted by the ‘ART women and children health’ working group from the National Agency of Biomedicine. Data were extracted from the French National Health System database (*Système National des Données de Santé—SNDS*) (> 99% of national deliveries). Mothers’ records were merged anonymously with those of their newborns and with previous pregnancy-related hospitalizations. Deliveries for which the mother and child’s data were not linked and multiple births were excluded. The final cohort included 93% of all French deliveries. We thus conducted a comparative analysis of the cohort of singleton births (deliveries ≥ 22 weeks of gestation and/or > 500 g of birthweight) which occurred in France over a 5-year period (2013–2017) and resulting from fresh embryo or frozen embryo transfers (fresh-ET or FET from IVF and ICSI cycles), IUI and NC. Follow-up data for this cohort were available until early childhood (mean 2.5 years old [1–5]). During this period, a total of 3,501,495 singleton births were identified (including 20,218 IUI, 45,303 fresh-ET, and 18,885 FET).

Data available in the hospitalization database were parity, plurality, maternal age, active smoking during pregnancy, obesity, maternal history of diabetes (type-1 or 2) and high blood pressure, the diagnosis of endometriosis, PCOS and POI, the mode of conception, the term, weight and sex of the newborn.

This database contains anonymized patient information and its access was legally approved.

### Children defects involving imprinted genes

Data were extracted from the national health database. Malformations were classified according to the WHO ICD-10 codes. We monitored several syndromes and diseases diagnosed on clinical features by paediatricians (and not genetic testing) but for which altered expression of imprinted genes are associated: Beckwith–Wiedemann (BWS), Silver–Russell (SRS), Prader–Willi (PWS), and Angelman (AS) syndromes, pseudohypoparathyroidism type Ib, neonatal diabetes mellitus (NDM), syndromes affecting the imprinted region of chromosome 14q32 (Temple and Kagami-Ogata syndromes), neuroblastoma and retinoblastoma (description of phenotypes and related imprinted genes is available in the Additional file 1: Table S1). In the following manuscript, we will refer to the syndromes by their names rather than the ICD-10 codes linked to them. Some children could have more than one disease diagnosed. For NDM newborns (characterized by hyperglycaemia in the neonatal period requiring insulin therapy and persisting for more than 2 weeks), we identified a subset of children who were prescribed medication or hospitalized for diabetes within one year after birth.

### Statistical analyses

Univariate and multivariate analyses were performed using logistic regression models to analyse the effect of ART conception and type of female infertility on the risk of imprinting disorders. Univariate analysis was used to compare the number of observed and expected cases (established from data of the NC group). Standardized incidence ratios [SIR] estimated from the control group of spontaneous conception according to the reproductive method were performed using a Poisson regression model. In multivariate analyses, we first assessed the effects of the mode of conception (ART [IUI/fresh-ET/FET] or NC) and other factors available in the hospitalization database that could potentially affect epigenetic risk. These factors were parity, maternal age (categorized in 10-year age groups), sex of the newborn, active smoking, obesity, history of diabetes (type 1 or 2), and maternal high blood pressure (HBP). Secondly, in our multivariate model, we included the type of female infertility (endometriosis, PCOS and POI), which provides estimates of the respective risk of the mode of conception and the type of female infertility adjusted for maternal parameters (age, primiparity, smoking, obesity, history of high blood pressure or diabetes), and newborn sex. Prematurity can potentially increase the incidence of NDM. However, our sensitivity analysis showed no noticeable differences in the magnitude of the risk estimates when premature infants were excluded from the analyses (data not shown), so all data were entered into a single database. The interactions between variables were tested, and we considered that a *p*-value < 0.05 provided evidence of a possible interaction. Adjusted odds ratios (aOR) and their 95% confidence intervals (CI) were estimated. Statistical analyses were performed using SAS 9.4 (SAS Institute Inc.). The significance level was set at a two-tailed *p*-value of < 0.05.

## Supplementary information

**Additional file 1: Table S1.** Syndromes and diseases involving imprinted genes including general features, molecular changes, cohort studies. **Table S2:** Risk of NDM according to mode of conception and type of female infertility in a subset of NDM newborns, i.e. infants who needed medications or were hospitalised for diabetes within 1 year after their birth. **Table S3:** Maternal characteristics according to the mode of conception.

## Abbreviations

ART: Assisted reproductive technologies
AS: Angelman syndrome
BWS: Beckwith–Wiedemann syndrome
FET: Frozen embryo transfers
Fresh-ET: Fresh embryo transfers
HBP: High blood pressure
ICSI: Intra-cytoplasmic injection (IVF with sperm injection)
IUI: Intrauterine insemination
IVF: In vitro fertilization
NC: Natural conception
NDM: Neonatal diabetes mellitus
PCOS: Polycystic ovary syndrome
POI: Primary ovarian insufficiency
PWS: Prader–Willi syndrome
SRS: Russell–Silver syndrome
TNDM: Transient NDM
