# Supplementary material for: Reproductive technologies, female infertility, and the risk of imprinting-related disorders
Source: Clin Epigenetics. 2020 Dec 11;12:191. doi: 10.1186/s13148-020-00986-3 (PMC7731556; doi:10.1186/s13148-020-00986-3)
Supplement: Supplementary file 1 — Additional file 1: Table S1. Syndromes and diseases involving imprinted genes including general features, molecular changes, cohort studies. Table S2: Risk of NDM according to mode of conception and type of female infertility in a subset of NDM newborns, i.e. infants who needed medications or were hospitalised for diabetes within 1 year after their birth. Table S3: Maternal characteristics according to the mode of conception. [file 13148_2020_986_MOESM1_ESM.docx]

**Supplementary Table S1: Syndromes and diseases involving imprinted genes including general features, molecular changes, cohort studies**

| **CIM10 codes** | **Disorders** |  | **Affected imprinted genes** | **Phenotypes** | **Prevalence in the general population -ORPHANET** |  | | **Previous cohort studies** | | |
| --- | --- | --- | --- | --- | --- | --- | --- | --- | --- | --- |
|  |  |  |  |  |  | **References** | **Percentage of ART/**  **Number of cases among ART population** | | **Countries** | **Database** |
| Q87.3 | Beckwith-Wiedemann syndrome |  | *IGF2, CDKN1C, KCNQ1OT1* (11p.15.5) | Overgrowth, anterior abdominal  wall defects, tumour and cancer predisposition, macroglossia, etc | 1-5:10,000 | Doornbos et al. 2007  Hiura et al. 2006  Lidegaard et al. 2005  Gicquel et al. 2003  Bowdin et al. 2007  Sutcliffe et al. 2006  DeBaun et al. 2003  Maher et al. 2003  Källén et al. 2005 | % of ART/BWS: 8.5%, RR=4.0 but NS after correction for impaired fertility  % of ART/BWS: 8.6%, RR=10  BWS/ART: 0/6,052, NS  % of ART/BWS: 4%, RR=3.2  BWS/ART: 1/2,492, NS  % of ART/BWS: 2.9%, RR=9.5, RR=3.6*  % of ART/BWS: 4.6%, RR= 6  % of ART/BWS: 4%, RR= 1.7  BWS/ART: 0/16,280, NS | | Netherlands  Japan  Denmark.  France  Ireland and central England  England/Ireland  USA  England  Sweden | Questionnaires  Questionnaires  National IVF and birth registries  National BWS registry  Questionnaires (2 IVF centres)  Questionnaires  Questionnaires  Birmingham BWS registry  National birth and congenital malformations registries |
| Q87.1 | Silver-Russell syndrome |  | *IGF2/H19*, *CDKN1C, MEST*, *GRB10*  upd(7) mat (7q32, 11p15) | Asymmetry, growth failure, adult-onset diseases, facial features, etc | 1-9:1,000,000 | Hiura et al. 2006  Lidegaard et al. 2005  Källén et al. 2005 | % of ART/SRS: 9.5%, RR=10  SRS/ART: 0/6,052, NS  SRS/ART: 1/16,280, NS | | Japan  Denmark  Sweden | Questionnaires  National IVF and birth registries  National birth and congenital malformations registries |
| Q87.1 | Prader-Willi syndrome |  | Various imprinted genes on chromosome 15, upd(15)mat  (15q11-q13) | Hypotonia, infertility, mental disorders, etc | 1-9:100,000 | Doornbos et al. 2007  Hiura et al. 2006  Lidegaard et al. 2005  Sutcliffe et al. 2006  Källén et al. 2005 | % of ART/PWS: 4.7% RR=2.2 but NS after correction for impaired fertility  % of ART/PWS: 1.5%, NS  PWS/ART: 0/6,052, NS  % of ART/PWS: 2%, NS  PWS/ART: 1/16,280, NS | | Netherlands  Japan  Denmark  England/Ireland  Sweden | National PWS registry  National PWS registry  National IVF and birth registries  Questionnaires  National birth and congenital malformations registries |
| Q93.5 | Angelman syndrome |  | *UBE3A,* upd(15)pat  (15q11-q13) | Abnormal behaviour with excessive laughter, intellectual disability, ataxia, etc | 1-9:100,000 | Doornbos et al. 2007  Hiura et al. 2006  Bowdin et al. 2007  Sutcliffe et al. 2006 | % of ART/AS: 6.3% RR=3.0 but NS after correction for impaired fertility  % of ART/AS: 1.6%, NS  AS/ART: 0/2492, NS  % of ART/AS: 0.8%, NS | | Netherlands  Japan  Ireland and central England  England/Ireland | Questionnaires  National AS registry  Questionnaires (2 IVF centres)  Questionnaires |
| E20.1 | Pseudohypoparathyroidism |  | Maternal transmission of inactive *GNAS* mutations, upd(20) pat (20q13.2) | Resistance to parathyroid hormone, obesity, osteodystrophy | 1-9:1,000,000 | Only case report studies (Fernandez et al. 2017; Goel et al. 2018) |  | |  |  |
| P70.2 | Transient  Neonatal Diabetes Mellitus (TNDM1) |  | *PLAGL1, HYMAI*, (11p15.1) paternal duplication of 6q24, upd(6) pat (6q24) | Diabetes, congenital abnormalities | Unknown in Orphanet | Sutcliffe et al. 2006 | % of ART/TNDM : 3%, NS | | England/Ireland | Questionnaires |
| Q99.8 | Temple syndrome [upd(14)mat] |  | (affecting the chromosome 14q32 imprinted region) : impact on *IGF2* expression through maternal RNAs of chromosome 14 | Growth retardation, hypotonia, obesity, mental disorders, etc | <1:1,000,000 | ND |  | |  |  |
| Q99.8 | Kagami-Ogata syndrome  [upd(14)pat] |  | (affecting the chromosome 14q32 imprinted region) | Polyhydramnios, developmental delay, placentomegaly, intellectual disability | <1:1,000,000 | ND |  | |  |  |
| C74.9 | Neuroblastoma |  | *DLK1-MEG3* imprinted domain | Clinical presentation is variable and depends on the stage and location of the tumor, which can develop at any site in the sympathetic nervous system (≈80% in the abdomen). | 1-5:10,000 | Spector et al. 2019  Williams et al. 2018  Reigstad et al. 2016  Lerner-Geva et al. 2016  Sundh et al. 2014  Williams et al. 2013 | % of ART/childhood neuroblastoma: 7.4%, NS  Neuroblastoma/donor ART: ND, NS  % of ART/childhood neuroblastoma: 2.1%, NS  % of ART/childhood neuroblastoma: 5.2%, NS  % of ART/childhood neuroblastoma: 18.7%, NS  % of ART/childhood neuroblastoma: ND, NS | | USA  England, Wales, Scotland Norway  Israel  Sweden, Denmark, Finland and Norway  England/Wales/Scotland | Registries of 14 states  National registries  National registry  National registry  National registries  National registries |
| C69.2 | Retinoblastoma |  | *RB1* (13q14).  Imprinted expression of *RB1* is linked to a differentially methylated CpG island in intron 2 of this gene (CpG 85) | Early clinical signs: leukocoria and strabismus then rapid progression towards loss of vision in the affected eye. | Unknown in Orphanet  1:17,000** | Spaan et al. 2019  Spector et al. 2019  Williams et al. 2018  Reigstad et al. 2016  Lerner-Geva et al. 2016  Sundh et al. 2014  Williams et al. 2013  Hargreave et al. 2013  Källén et al. 2010  Marees et al. 2009 | % of ART/childhood retinoblastoma: 100%, NS  % of ART/childhood retinoblastoma: 6.7%, NS  Retinoblastoma/donor ART: ND, NS  % of ART/childhood retinoblastoma: 1%, NS  % of ART/childhood retinoblastoma: 18.1%, RR=7.8  % of ART/childhood retinoblastoma: 11.4%, NS  % of ART/childhood retinoblastoma: ND, NS  % of ART/childhood retinoblastoma: ND, NS  % of ART/childhood retinoblastoma: 3.4%, NS  % of ART/childhood retinoblastoma: 3.4%, RR=2.5 | | Netherlands  USA  England/Wales/Scotland Norway  Israel  Sweden, Denmark, Finland, Norway  England/Wales/Scotland  Denmark  Sweden  Netherlands | Voluntary national registry  Registries of 14 states  National registry  National registry  National registry  National registries  National registries  National registry  National birth and congenital malformations registries  Questionnaires |

ND: not documented; NS: not significant; Upd()mat: uniparental disomy (chromosome affected) maternally inherited; Upd()pat: uniparental disomy (chromosome affected) paternally inherited

*RR with correction for non-responders

**Moll et al. 1997

([Bowdin, et al., 2007](#_ENREF_1), [DeBaun, et al., 2003](#_ENREF_2), [Doornbos, et al., 2007](#_ENREF_3), [Fernandez, et al., 2017](#_ENREF_4), [Gicquel, et al., 2003](#_ENREF_5), [Goel, et al., 2018](#_ENREF_6), [Hargreave, et al., 2013](#_ENREF_7), [Hiura, et al., 2012](#_ENREF_8), [Kallen, et al., 2010](#_ENREF_9), [Kallen, et al., 2005](#_ENREF_10), [Lerner-Geva, et al., 2017](#_ENREF_11), [Lidegaard, et al., 2005](#_ENREF_12), [Maher, et al., 2003](#_ENREF_13), [Marees, et al., 2009](#_ENREF_14), [Moll, et al., 1997](#_ENREF_15), [Reigstad, et al., 2016](#_ENREF_16), [Spaan, et al., 2019](#_ENREF_17), [Spector, et al., 2019](#_ENREF_18), [Sundh, et al., 2014](#_ENREF_19), [Sutcliffe, et al., 2006](#_ENREF_20), [Williams, et al., 2018](#_ENREF_21), [Williams, et al., 2013](#_ENREF_22))

**Supplementary Table S2: Risk of NDM according to mode of conception and type of female infertility in a subset of NDM new-borns, i.e. infants who had needed medications or were hospitalised for diabetes within one year after their birth**

|  | **Children with NDM** | |  |
| --- | --- | --- | --- |
|  | **N** | **%** | ***p**** |
| Mode of conception |  |  |  |
| NC | 2,167 | 0.06 |  |
| Fresh-ET | 38 | 0.08 | 0.04 |
| FET | 13 | 0.07 | 0.32 |
| IUI | 13 | 0.06 | 0.41 |
| Female infertility |  |  |  |
| Endometriosis |  |  |  |
| No | 2,199 | 0.06 |  |
| Yes | 32 | 0.09 | 0.04 |
| PCOS |  |  |  |
| No | 2,224 | 0.06 |  |
| Yes | 7 | 0.10 | 0.08 |
| POI |  |  |  |
| No | 2,228 | 0.06 |  |
| Yes | 3 | 0.21 | 0.01 |

Fresh-ET : fresh embryo transfer, FET : frozen embryo transfer, IUI : intra-uterine insemination, NC : natural conception, NDM: neonatal diabetes mellitus, PCOS: polycystic ovary syndrome, POI: primary ovarian insufficiency

*Univariate Poisson regression analysis

**Supplementary Table S3: Maternal characteristics according to the mode of conception.**

|  | NC | | Fresh-ET | | FET | | IUI | | | | *P** |
| --- | --- | --- | --- | --- | --- | --- | --- | --- | --- | --- | --- |
|  | **N** | **%** | **N** | **%** | **N** | **%** | **N** | | | **%** |  |
| Total | 3,417,089 |  | 45,303 |  | 18,885 |  | | 20,218 | |  |  |
| Mean maternal age –y (std) | 29.9 (5.3) |  | 33.2 (4.3) |  | 33.4 (4.3) |  | | 32.6 (4.4) | |  |  |
|  |  |  |  |  |  |  | |  | |  | <0.0001 |
| <20 | 79,617 | 2.33 | 6 | 0.01 | 1 | 0.01 | | 2 | | 0.01 |  |
| 20-29 | 1,556,674 | 45.56 | 9,459 | 20.88 | 3,503 | 18.55 | | 5,139 | 25.42 | |  |
| 30-39 | 1,649,883 | 48.28 | 32,124 | 70.91 | 13,740 | 72.76 | | 13,675 | 67.64 | |  |
| >=40 | 13,0915 | 3.83 | 3,714 | 8.20 | 1,641 | 8.69 | | 1,402 | 6.93 | |  |
| Primiparity | 1,369,652 | 40.08 | 29,431 | 64.96 | 10,430 | 55.23 | | 12,843 | 63.52 | | <0.0001 |
| Obesity | 153,067 | 4.48 | 1,722 | 3.8 | 756 | 4 | | 1,029 | 5.09 | | <0.0001 |
| Maternal smoking | 145,793 | 4.27 | 956 | 2.11 | 355 | 1.88 | | 419 | 2.07 | | <0.0001 |
| History of HBP | 22,614 | 0.66 | 392 | 0.87 | 196 | 1.04 | | 156 | 0.77 | | <0.0001 |
| History of diabetes | 22,797 | 0.67 | 368 | 0.81 | 142 | 0.75 | | 179 | 0.89 | | <0.0001 |

HBP: high blood pressure, OR: odds ratio, std: standard deviation, y: years.

*Chi2 test.

**References**

Bowdin S, Allen C, Kirby G, Brueton L, Afnan M, Barratt C, Kirkman-Brown J, Harrison R, Maher ER, Reardon W. A survey of assisted reproductive technology births and imprinting disorders. *Hum Reprod* 2007;22: 3237-3240.

DeBaun MR, Niemitz EL, Feinberg AP. Association of in vitro fertilization with Beckwith-Wiedemann syndrome and epigenetic alterations of LIT1 and H19. *Am J Hum Genet* 2003;72: 156-160.

Doornbos ME, Maas SM, McDonnell J, Vermeiden JP, Hennekam RC. Infertility, assisted reproduction technologies and imprinting disturbances: a Dutch study. *Hum Reprod* 2007;22: 2476-2480.

Fernandez M, Zambrano MJ, Riquelme J, Castiglioni C, Kottler ML, Juppner H, Mericq V. Pseudohypoparathyroidism type 1B associated with assisted reproductive technology. *J Pediatr Endocrinol Metab* 2017;30: 1125-1132.

Gicquel C, Gaston V, Mandelbaum J, Siffroi JP, Flahault A, Le Bouc Y. In vitro fertilization may increase the risk of Beckwith-Wiedemann syndrome related to the abnormal imprinting of the KCN1OT gene. *Am J Hum Genet* 2003;72: 1338-1341.

Goel NJ, Meyers LL, Frangos M. Pseudohypoparathyroidism type 1B in a patient conceived by in vitro fertilization: another imprinting disorder reported with assisted reproductive technology. *J Assist Reprod Genet* 2018;35: 975-979.

Hargreave M, Jensen A, Deltour I, Brinton LA, Andersen KK, Kjaer SK. Increased risk for cancer among offspring of women with fertility problems. *Int J Cancer* 2013;133: 1180-1186.

Hiura H, Okae H, Miyauchi N, Sato F, Sato A, Van De Pette M, John RM, Kagami M, Nakai K, Soejima H *et al.* Characterization of DNA methylation errors in patients with imprinting disorders conceived by assisted reproduction technologies. *Hum Reprod* 2012;27: 2541-2548.

Kallen B, Finnstrom O, Lindam A, Nilsson E, Nygren KG, Olausson PO. Cancer risk in children and young adults conceived by in vitro fertilization. *Pediatrics* 2010;126: 270-276.

Kallen B, Finnstrom O, Nygren KG, Otterblad Olausson P, Wennerholm UB. In vitro fertilisation in Sweden: obstetric characteristics, maternal morbidity and mortality. *BJOG* 2005;112: 1529-1535.

Lerner-Geva L, Boyko V, Ehrlich S, Mashiach S, Hourvitz A, Haas J, Margalioth E, Levran D, Calderon I, Orvieto R *et al.* Possible risk for cancer among children born following assisted reproductive technology in Israel. *Pediatr Blood Cancer* 2017;64.

Lidegaard O, Pinborg A, Andersen AN. Imprinting diseases and IVF: Danish National IVF cohort study. *Hum Reprod* 2005;20: 950-954.

Maher ER, Brueton LA, Bowdin SC, Luharia A, Cooper W, Cole TR, Macdonald F, Sampson JR, Barratt CL, Reik W *et al.* Beckwith-Wiedemann syndrome and assisted reproduction technology (ART). *J Med Genet* 2003;40: 62-64.

Marees T, Dommering CJ, Imhof SM, Kors WA, Ringens PJ, van Leeuwen FE, Moll AC. Incidence of retinoblastoma in Dutch children conceived by IVF: an expanded study. *Hum Reprod* 2009;24: 3220-3224.

Moll AC, Kuik DJ, Bouter LM, Den Otter W, Bezemer PD, Koten JW, Imhof SM, Kuyt BP, Tan KE. Incidence and survival of retinoblastoma in The Netherlands: a register based study 1862-1995. *Br J Ophthalmol* 1997;81: 559-562.

Reigstad MM, Larsen IK, Myklebust TA, Robsahm TE, Oldereid NB, Brinton LA, Storeng R. Risk of Cancer in Children Conceived by Assisted Reproductive Technology. *Pediatrics* 2016;137: e20152061.

Spaan M, van den Belt-Dusebout AW, van den Heuvel-Eibrink MM, Hauptmann M, Lambalk CB, Burger CW, van Leeuwen FE, group OM-s. Risk of cancer in children and young adults conceived by assisted reproductive technology. *Hum Reprod* 2019;34: 740-750.

Spector LG, Brown MB, Wantman E, Letterie GS, Toner JP, Doody K, Ginsburg E, Williams M, Koch L, Schymura MJ *et al.* Association of In Vitro Fertilization With Childhood Cancer in the United States. *JAMA Pediatr* 2019;173: e190392.

Sundh KJ, Henningsen AK, Kallen K, Bergh C, Romundstad LB, Gissler M, Pinborg A, Skjaerven R, Tiitinen A, Vassard D *et al.* Cancer in children and young adults born after assisted reproductive technology: a Nordic cohort study from the Committee of Nordic ART and Safety (CoNARTaS). *Hum Reprod* 2014;29: 2050-2057.

Sutcliffe AG, Peters CJ, Bowdin S, Temple K, Reardon W, Wilson L, Clayton-Smith J, Brueton LA, Bannister W, Maher ER. Assisted reproductive therapies and imprinting disorders--a preliminary British survey. *Hum Reprod* 2006;21: 1009-1011.

Williams CL, Bunch KJ, Murphy MFG, Stiller CA, Botting BJ, Wallace WH, Davies MC, Sutcliffe AG. Cancer risk in children born after donor ART. *Hum Reprod* 2018;33: 140-146.

Williams CL, Bunch KJ, Stiller CA, Murphy MF, Botting BJ, Wallace WH, Davies M, Sutcliffe AG. Cancer risk among children born after assisted conception. *N Engl J Med* 2013;369: 1819-1827.
